# Plants under the Attack of Allies: Moving towards the Plant Pathobiome Paradigm

**DOI:** 10.3390/plants10010125

**Published:** 2021-01-09

**Authors:** Mohamed Mannaa, Young-Su Seo

**Affiliations:** Department of Integrated Biological Science, Pusan National University, Busan 46241, Korea; mannaa@pusan.ac.kr

**Keywords:** holobiome, meta-omics, pathobiome, phytobiome, symbiome

## Abstract

Plants are functional macrobes living in a close association with diverse communities of microbes and viruses as complex systems that continuously interact with the surrounding environment. The microbiota within the plant holobiont serves various essential and beneficial roles, such as in plant growth at different stages, starting from seed germination. Meanwhile, pathogenic microbes—differentiated from the rest of the plant microbiome based on their ability to damage the plant tissues through transient blooming under specific conditions—are also a part of the plant microbiome. Recent advances in multi-omics have furthered our understanding of the structure and functions of plant-associated microbes, and a pathobiome paradigm has emerged as a set of organisms (i.e., complex eukaryotic, microbial, and viral communities) within the plant’s biotic environment which interact with the host to deteriorate its health status. Recent studies have demonstrated that the one pathogen–one disease hypothesis is insufficient to describe the disease process in many cases, particularly when complex organismic communities are involved. The present review discusses the plant holobiont and covers the steady transition of plant pathology from the one pathogen–one disease hypothesis to the pathobiome paradigm. Moreover, previous reports on model plant diseases, in which more than one pathogen or co-operative interaction amongst pathogenic microbes is implicated, are reviewed and discussed.

## 1. Introduction

Like all other organisms, plants do not lead solitary lives, as there are myriads of microbes and viruses living around and within them. Some microbes, whether endophytic or epiphytic, play diverse roles in supporting healthy plant growth, whereas others are pathogenic, which can become dominant over the beneficial ones to cause disease [1]. In recent years, various cutting-edge tools developed for studying the associations between microbes and plants and extensive modern research on plant microbiomes have dramatically furthered our knowledge on the ecological functions and key roles of the plant microbiome in supporting plant adaptability to dynamic environments [2]. Currently, plant-associated microorganisms are considered reservoirs of additional genes and traits, which are critical to the growth and development of the host [3]. Furthermore, the plant pathobiome—which represents the disease-causing agents in the context of the interaction between the microbial communities and plant host in its biotic environment—is another important component of the plant microbiome that remains relatively understudied [2].

Research focussing on the widely accepted one pathogen–one disease hypothesis has led to many breakthroughs, such as the identification of diseases and novel disease-causing organisms, as well as the development of control strategies using effective compounds against individual pathogens, which have proven successful in controlling several diseases [4]. However, this came at the cost of neglect of plant pathology in a holistic approach—or systems-based plant pathology—in which communities and their interactions are considered rather than individual organisms. This reductionist scheme has limited our ability to overcome certain important challenges, such as the emergence of novel and severe diseases, with little that could be done to counter these diseases without considering the associated biotic factors [4].

In this review, the concept of plant holobiont is introduced in the light of recent advances in meta-omics analyses based on next-generation sequencing techniques, which have paved the way for gaining a comprehensive knowledge of the diverse roles of microbes in supporting healthy plant growth or causing diseases. According to the plant pathobiome paradigm, the one pathogen–one disease hypothesis is not sufficient to explain the disease process in several cases, as emerging evidence has indicated that complex pathogenic communities, referred to as the pathobiome, contribute to disease development. Moreover, case studies of plant diseases examined from the viewpoint of pathobiome are discussed and future opportunities reviewed.

## 2. Plant Holobiont

As functional macrobes living in a close association with diverse communities of microbes and viruses, plants should be considered a ‘holobiont’, viewed as a complex system in continuous interaction with the resident microbes and the surrounding environment [5]. The microbes with their functional genes represent the plant microbiome, or the phytobiome, and their composition may differ among individual plants, as well as across various stages of growth or sites and tissues of the same plant. Despite the extensive taxonomic overlap between the microbiomes of different plant tissues, each compartment exhibits a unique composition of strains and species, as evidenced from the specificity of different operational taxonomic units (OTUs) in various tissues of plants within the same genus [3,6].

The beneficial roles of microbes associated with plants include, but are not limited to, supporting plant growth at different stages starting from seed germination, promoting plant resistance to biotic and abiotic stresses, and assisting plants in nutrient uptake [7,8,9]. The plant growth-promoting bacteria and the arbuscular mycorrhiza represent the beneficial microbes that are mostly involved in supporting plant growth and nutrition by facilitating nutrients mobilisation. The mycorrhizae were even reported to manipulate plant hormonal signalling to facilitate their colonisation of the root surface in a way similar to the mechanisms of some pathogenic microbes, while in this case, the hijacking is beneficial for the host plant [9].

Alternatively, pathogenic microbes are also a part of the phytobiome, although this concept has been partly overlooked in most studies on this topic [3]. Despite their presence within the microbial communities, pathogenic microbes are differentiated from the rest of the phytobiome based on their ability to damage the plant tissues through transient blooming under specific conditions, which is consistent with the core concept of the disease triangle in epidemiology [10].

Defining specific taxonomic groups as pathogenic or beneficial could be misleading, as some microbial genera might include beneficial members that support growth at certain stages of a plant species, but are pathogenic at another stage or to other plant species. For instance, while some members of the genus *Rhizoctonia* are essential for promoting seed germination and supporting the growth of certain orchid species, others are devastating pathogens causing seedling damping-off, root rot, stem rot, and canker in several plants and even post-harvest rot in some crops [11,12]. Therefore, studying the types and taxonomic composition of plant microbiomes might not be sufficient to completely understand the roles of the plant microbiome, and the functional potential of the characterised microbial structure must be investigated within their communities.

Studies have shown that under specific conditions, the stable, beneficial plant microbiome may be altered to facilitate the development and establishment of certain diseases. A model representing this phenomenon is the olive knot disease caused by *Pseudomonas savastanoi* pv. *Savastanoi*. The knots formed by *Pseudomonas savastanoi* pv. *Savastanoi* in the aerial parts of olive trees harbour a specific multi-species community of endophytic non-pathogenic bacteria, which co-operate with the main causative bacteria to enhance disease severity [13,14]. The well-documented co-existence and shared quorum-sensing signals of specific bacterial communities of *Pantoea* and *Erwinia* in the olive knots and the causative agent *Pseudomonas savastanoi* pv. *Savastanoi* in different olive-growing regions of the world suggests the co-evolution and conserved roles of this bacterial consortium in promoting disease development [15]. Co-inoculation of *Pantoea* and *Erwinia* species with *Pseudomonas savastanoi* pv. *Savastanoi* facilitated bacterial colonisation, nutrient exploitation, plant defence disruption, and knot enlargement [13,16].

In this context, plant pathogenic microbes may specifically manipulate the structure of the plant microbiome to generate conditions conducive to their own survival and colonisation. Kim et al. [17] demonstrated that the plant pathogen *Burkholderia glumae* employs the specific type-6 secretion system (T6SS) for interaction with rice endophytic microbes, thereby reducing the populations of specific bacterial genera, such as *Luteibacter* and *Dyella*, which promote plant growth and contribute to protection against pathogenic bacteria. Metagenomic analysis in their study also revealed significant changes in the community structure of endophytic microbiota in infected rice plants compared with non-infected plants or plants infected with a T6SS-defective *B. glumae* mutant. Specifically, these changes facilitated the colonisation and establishment of *B. glumae* at the early stages of infection [17].

Another example in which the plant-associated beneficial bacteria turn harmful under specific conditions to support the development of the disease is the root-knot caused by the nematode *Meloidogyne incognita*. Nematode infection is associated with the presence of specific microbes harbouring abundant genes involved in pathogenesis, such as genes encoding plant polysaccharide-degrading enzymes [18]. Hence, assessments of the taxonomic composition should always be aligned to functional analyses of the existing microbial communities, and the plant holobiont should be separated into the symbiome or pathobiome under specific conditions based on function rather than taxonomy.

**Figure 1:**
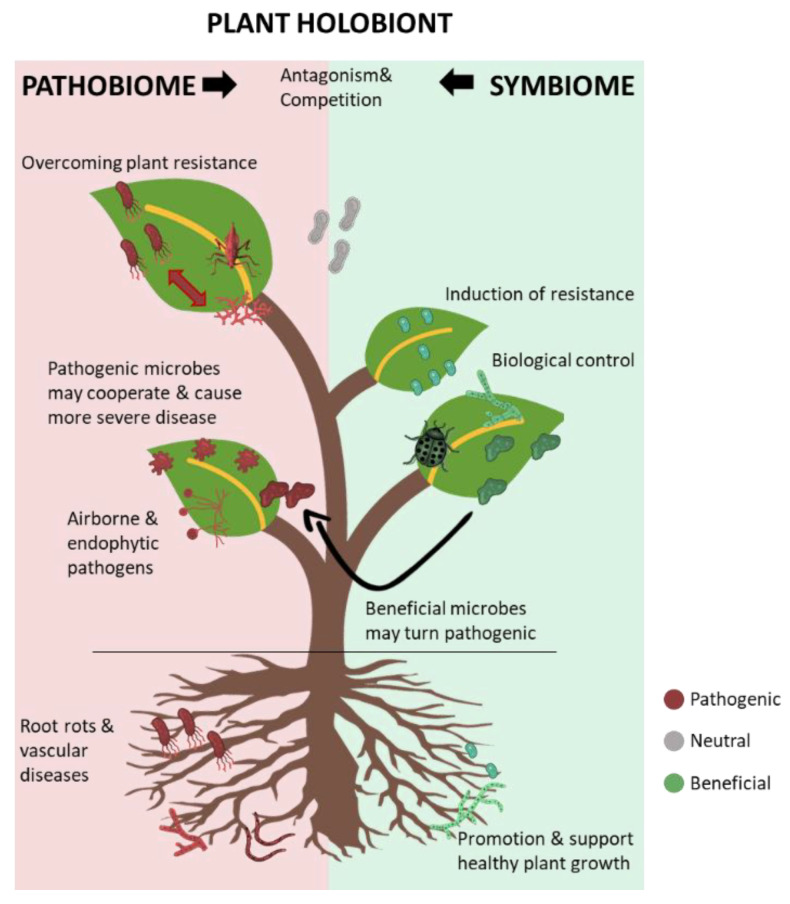
A schematic diagram of the plant holobiont as a complex system, including beneficial plant-associated microbes—the symbiome—and the collective pathogenic microbes—the pathobiome. The beneficial and pathogenic microbes are in continuous antagonism and competition for space and nutrients. When pathogenic microbes prevail, they interfere with normal plant functions, leading to disease development. Co-operative interactions amongst pathogenic microbes may result in more severe disease and multiple infections. The stable, beneficial plant microbiome may be altered to facilitate the development of certain diseases, as observed in the olive knot disease.

Overall, the plant holobiont could be represented as a never-ending war between the allies of pathogenic microbes, as the pathobiome, and the key beneficial microbes, as the symbiome. A representation of the plant holobiont is shown in Figure 1.

## 3. Meta-Omics and Plant Phytobiome Studies

The availability of molecular tools enabling the profiling of the exact structure of the microbiome without the need to culture the microbes has revolutionised our understanding of various ecosystems, including plants. In recent years, there have been remarkable advances in next-generation sequencing-based meta-omics, serving as a magnifying lens to unveil the structure and functions of the plant-associated microbial communities [19]. Currently available high-throughput sequencing approaches, such as the Illumina (e.g., HiSeq and MiSeq), Roche 454 GS FLX+, and Ion Torrent/Ion Proton platforms, have rapidly advanced and have been widely used in recent studies of plant-associated microbes [20]. In the past, studies on microbial communities mostly relied on the PCR amplification of a taxonomically relevant genomic marker, such as the hypervariable regions of the 16S rRNA gene to examine the bacterial diversity or the ITS region to examine the fungal diversity and taxonomic classification of the existing microbes. However, such metabarcoding techniques relying on the amplification of a single gene have some shortcomings related to inherent sequencing errors associated with incorrectly assembled amplicons. Moreover, the amplicons are short, and PCR-associated biases fail to accurately capture the community diversity [21]. More importantly, amplicon sequencing provides insights into the taxonomic composition of microbial communities in terms of OTUs alone, and the biological functions of the identified taxa cannot be directly determined, although phylogenetic reconstruction can offer some insights into these functions [22]. Alternatively, shotgun metagenomic sequencing of uncultured microbiomes does not require the amplification of specific genomic loci, and instead, whole DNA is sheared into small fragments and subsequently sequenced. Regardless of the high complexity and massive metagenomic data requirement, this approach can retrieve taxonomic information and infer potential biological functions of the identified taxa [23].

Other reliable approaches to study the functions of the plant-associated microbial communities are meta-transcriptomic and meta-proteomic analyses, which can also reveal the effects of the environment or the host plant genotype on the functions of the microbial communities [24]. These analyses can serve as useful tools to assess the abundance and functions of transcripts under specific conditions to infer whether these conditions trigger beneficial features, such as disease-suppressive activities, reflected by the high abundance of transcripts promoting plant growth, resistance, xenobiotic production, and pesticide-degrading enzyme secretion, or pathogenic features, reflected by the high abundance of transcripts promoting virulence factors, such as plant cell wall-degrading enzymes or phytopathogen effectors of the avrE family [24,25,26]. These analyses can also be useful to clarify the plant response to microbial activity; as such, the transcriptomic analysis may offer insight into plant host genes possibly manipulated by the action of pathogens in a disease setting, such as the downregulation of photosynthesis-related genes, consistent with the development of specific disease symptoms, or the upregulation of genes involved in other physiological processes [27,28].

## 4. Metabolomics and Modern Plant Pathology

Studying the metabolites of both plants and their associated microbes represents another important approach to understand the processes of healthy growth and disease development in plants. Classic plant pathology focuses on studying the importance of individual metabolites in plant diseases; however, in recent years, substantial technical developments have enabled a more holistic approach to study metabolomics at the global level [29]. From the perspective of pathogens, metabolomics facilitates the understanding of mechanisms through which pathogens may overcome plant immunity and invade the plants. From the perspective of plants, metabolomics facilitates the understanding of physiological changes, including reinforcement of the plant cell wall or plant metabolism (e.g., the production of reactive oxygen species, plant hormones, antimicrobial, and signalling molecules) in response to pathogenic attack [30,31,32]. Several studies have confirmed the utility of metabolomics in understanding the plant response to disease. Metabolomic analysis of soybean infected with *Phytophthora sojae* indicated that specific metabolites and sugars were accumulated in resistant plants as opposed to those in susceptible plants, suggesting the involvement of these molecules in plant defence or resistance [33]. Metabolomic analysis of citrus canker in transgenic plants revealed that the production of the antimicrobial peptide sarcotoxin offered protection against the pathogen and induced oxidative stress response [34].

Similar studies have indicated the utility of metabolomic analyses in understanding pathogenesis, such as mechanisms through which pathogens overcome plant defence and colonise plant tissues. Detection of a series of plant and pathogen metabolites, such as molecules related to infection or amino acids and sugars induced upon pathogen attack to promote pathogen growth, has contributed to unveiling the plant–microbe interactions in pathogenesis [29,35]. Several recent metabolomic studies have detected such metabolites with pivotal roles in pathogenesis. Low-molecular-weight organic compounds from the root exudates of potato were shown to stimulate the germination of the resting spores and the release of more zoospores from *Spongospora subterranea*, the causative agent of powdery scab [36]. In other studies, certain pathogens were shown to deliberately manipulate the host plant metabolism and consequently induce disease symptoms. Meta-transcriptomic and metabolomic analyses have been used to reveal the role of *Plasmodiophora brassicae*, the causative agent of clubroot in Brassicaceae, in regulating cytokinin metabolism during gall formation in infected plants [37]. Another clear example of pathogens manipulating or regulating the host plant metabolism for their own benefits has been reported in a metabolomic study of the biotrophic pathogen *Gymnosporangium asiaticum*, the causative agent of rust disease; this pathogen alters the host metabolite levels, resulting in the accumulation of tetrose and pentose sugar alcohols, which disrupt important plant functions, such as cell wall synthesis and lesion repair, ultimately developing parasitic symptoms [38].

Metabolomics is useful to describe the mechanisms through which pathogens cause disease by analysing the pathogen-produced effectors during infection. Typically, such effectors are small proteins, but several non-proteinaceous effectors have been identified, such as fungal chemical secondary metabolites and small RNAs (sRNAs), which perform vital virulence-related functions [39]. In addition to their roles as toxic effectors, fungal secondary metabolites are involved in pathogen colonisation in plant tissues [40]. Previous transcriptomic studies have reported the expression or upregulation of numerous biosynthetic gene clusters of secondary metabolites in phytopathogens, such as *Pyricularia oryzae*, *Colletotrichum higginsianum*, *Zymoseptoria tritici*, and *Fusarium graminearum*, at specific early stages of plant colonisation [39,41,42]. Moreover, sRNAs from fungal phytopathogens have been reported to inhibit the expression of plant immunity-related genes through transcriptional repression [43]. *Botrytis cinerea* uses sRNA to hijack the RNA interference machinery of the host plant by binding to the argonaute 1 protein and producing an RNA-induced silencing complex [43]. Other studies have reported similar bidirectional cross-kingdom RNA interference of plant immunity genes in phytopathogenic fungi, such as *Verticillium dahliae* and *Puccinia striiformis* f. sp. *Tritici* [44,45].

**Figure 2:**
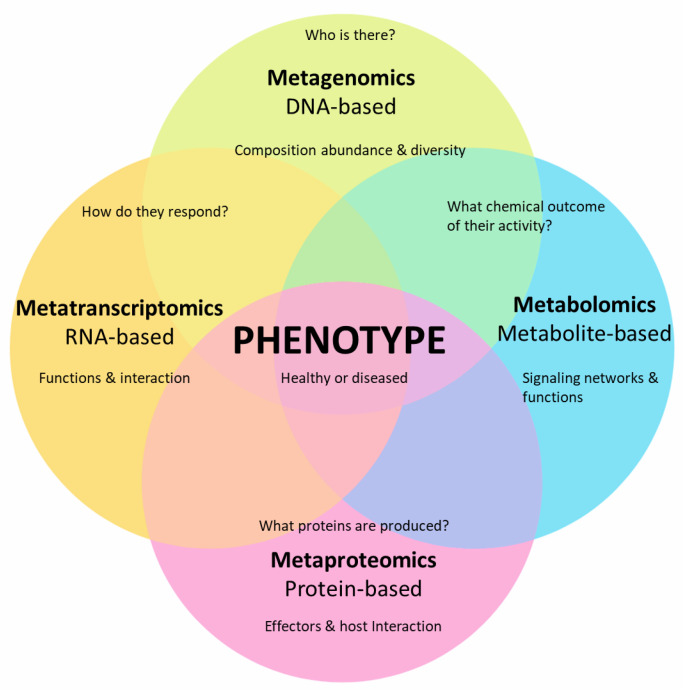
Advances in various aspects of meta-omics (i.e., meta-genomics, meta-transcriptomics, and meta-proteomics) in combination with metabolomics have substantially improved our understanding of plant-associated microbial communities and their influence on the phenotype (healthy plant growth or disease development).

Collectively, meta-omics (i.e., meta-genomics, meta-transcriptomics, and meta-proteomics) combined with metabolomics has substantially furthered our knowledge of plant microbiome and plant–microbe interactions at the molecular level through approaches, such as the detection of microbial types and the expression of genes, proteins, and metabolites (Figure 2).

## 5. Plant Pathobiome

The concept of pathobiome originally emerged from research on the human microbiome, which was found to be essential for sustaining human health. The dysbiosis of such a balanced, diverse, and rich community structure is always linked to an unhealthy status, exposing the gut to pathogenic infections and other metabolic disorders. Accordingly, the term pathobiome was coined to describe the overall disease-related microbial community [46]. The concept of pathobiome has also been used to represent the complex pathogenic organisms affecting animals and plants [47]. In general, a healthy plant is closely associated with a stable and diverse community of organisms, representing the biotic factors that support plant growth and serve important functions for the host—which are together described as the symbiome. A collective shift of this ecologically stable symbiome to the pathobiome involves a compositional transition, leading to the disruption of normal growth such that the plants cannot perform functions to the best of their genetic potential [48]. Although there is an inconsistency in the definition of pathobiome in the literature, it could simply be defined as the set of organisms (i.e., complex eukaryotic, microbial, and viral communities) within the plant’s biotic environment, which interact with the host to deteriorate its health status [47].

Although the concept of pathobiome is relatively new to plant pathology and have originated following the recent advancements in the multi-omics approaches, several early studies have investigated the occurrence of diseases with multi-species of causal agents and mixed infections which were referred as “disease complex” [15,16]. Several examples from previous studies where a complex of bacterial or fungal species are involved in the disease process, such as the tomato pith necrosis, broccoli soft rot and young grapevine decline where more than one bacterial or fungal species are in synergistic interaction and implicated in the disease development [49,50,51,52].

Recent plant pathological studies have demonstrated that the one pathogen—one disease hypothesis based on the fundamental Koch’s postulates is insufficient to describe the disease process in a more holistic and realistic way, particularly when complex communities of organisms are involved [24]. Even when a single pathogenic agent is implicated, other accompanying organisms are likely to mitigate or enhance the pathogenic effects and should thus be considered a part of the disease process [1,53]. Previous studies have reported diverse co-operative interactions amongst pathogenic microbes, which result in the promotion of growth of the involved pathogenic agents and consequent increase in the disease severity.

### 5.1. Co-Infections by and Interactions amongst Pathogenic Agents

In a previous study, a co-operative interaction between the seed-borne pathogenic rice bacterium *Burkholderia glumae*, the causative agent of panicle and seedling blight, and the airborne pathogenic fungus *Fusarium graminearum* was observed; as such, both pathogens positively affected the dispersal and survival of each other, as well as promoted disease progression [53]. The study demonstrated that both organisms have co-evolved to adapt and maximise the benefits of such a co-existence on rice as a host, even though one is seed-borne and the other is airborne. *Fusarium graminearum* produced abundant spores and toxins and was resistant to toxoflavin, the key virulence factor with antifungal activity produced by *Burkholderia glumae*. The colonisation and competitive ability of *Fusarium graminearum* were also promoted by *Burkholderia glumae*, as toxoflavin produced by the bacterium suppressed other competitor fungi in favour of the allied attacker *Fusarium graminearum*. Meanwhile, *Burkholderia glumae* could physically attach to the *Fusarium graminearum* conidia, which offered protection from UV-induced damage and facilitated spread through aerial dispersal [53]. Previous studies have also reported the beneficial interaction and close association between *Burkholderia* sp. And other fungi, as well as the ability of the bacterium to establish a close association with the fungus to utilise fungal-secreted metabolites for its own benefit [54].

Another example of the evolution of this unique tripartite (bacteria–fungi–plant) system for making an allied effort to cause disease is the bacterial endosymbionts of plant pathogenic fungi [55]. For over two decades, the zygomycete fungus *Rhizopus microsporus* was thought to be the sole causative agent of rice seedling blight and producer of the major virulence factor rhizotoxin, which effectively binds to the β-tubulin of eukaryotic cells and inhibits mitosis in the roots of rice seedlings [56,57]. Intriguingly, rhizotoxin was then confirmed to be biosynthesised by a bacterium residing within the fungal cytosol, proving that it is in fact not a fungal metabolite [58]. The fungal obligate endosymbiont *Mycetohabitans rhizoxinica* (formerly known as *Burkholderia rhizoxinica*) was isolated from the fungus, and the rhizotoxin biosynthesis gene cluster was characterised, confirming its inevitable role in causing the disease and establishing the bacterium as another etiological agent along with its host fungus *Rhizopus microsporus* [57,58]. The host fungus metabolism and vegetative reproduction were strictly dependent on the endosymbiotic bacterium, which also provided chemical weapons, and in turn, received shelter and nutrients from the fungus [59]. Moreover, the endosymbiont-free fungus, treated with antibiotics, was unable to sporulate. This dependence ensures a strong, unbreakable alliance and continued co-existence of both organisms as a phytopathogenic unit [59,60].

Plant pathogens could also co-operate by suppressing plant innate immunity, paving the way for subsequent secondary infections by other pathogens to which the plants are naturally resistant, and plants would not be infected by them unless the plant defence is initially broken by the first striker. The biotrophic oomycete *Albugo candida* causes broad-spectrum suppression of defence in wild and domesticated crucifer hosts (i.e., *Arabidopsis thaliana* and *Brassica juncea*), resulting in enhanced susceptibility to infection by several pathogens causing downy or powdery mildew [61]. In a comprehensive study investigating these suppressive mechanisms, *Albugo* spp. were found to suppress plant defence in *Arabidopsis thaliana* through physiological changes in resistance-related tryptophan-derived secondary metabolite biosynthesis and salicylic acid-mediated plant defence to facilitate infection and complete colonisation of *Phytophthora infestans* [62].

Plant pathogens sharing the host plants may also interact in an antagonistic way and compete for colonisation of the affected tissues. Three genetically related pathogenic *Burkholderia* species, namely, *Burkholderia glumae*, *Burkholderia gladioli*, and *Burkholderia plantarii*, causing rice seedling and panicle blight have been reported to co-exist on rice plants, and their interactions have been previously studied. *Burkholderia gladioli* exerted a strong antagonistic activity against both *Burkholderia glumae* and *Burkholderia plantarii*, as demonstrated by in vitro experiments and *in planta* assays [63]. Consistent with these results, the antagonistic activity of *Burkholderia gladioli* against other bacterial species, including the related rice pathogenic *Burkholderia* species, was reported in several other studies [64,65,66]. Hence, pathogenic organisms do not always interact in a co-operative manner to promote the disease and can also interact in an antagonistic manner. Examples of the different types of interactions among plant pathogenic fungal and bacterial species are summarised in Table 1.

**Figure 3:**
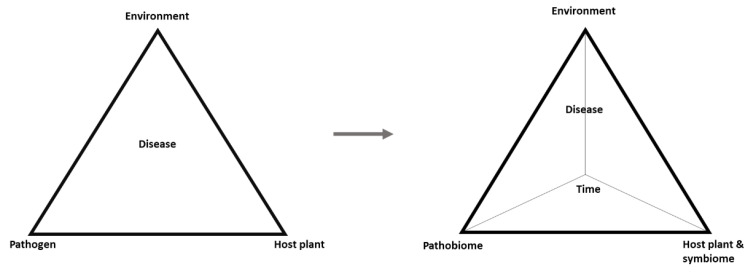
Plant pathology is currently witnessing a steady transition from the classical three-dimensional disease triangle to a more holistic view to explain plant disease through a four-dimensional disease pyramid, including time as the fourth dimension, representing the dynamics of the factors involved, and the plants are supported by the symbiome (beneficial organisms), reflecting a paradigm shift from the one pathogen–one disease hypothesis to the pathobiome concept.

In this light, understanding the pathobiome requires a deeper knowledge of the types of organisms involved, the influence they have on one another, the survival and transmission of the pathogens, and the biotic and abiotic factors that may affect the pathobiome and pathogenesis [2]. These points represent a research challenge for ongoing and future studies. Nonetheless, a transition is evident from the classic disease triangle comprising the pathogen, host, and environment to a more realistic disease pyramid in which pathogens within their community are considered as the pathobiome, the host is supported by its symbiome, and time is added as the fourth dimension representing dynamics of the other factors (Figure 3).

### 5.2. Development and Assembly of the Pathobiome

Microorganisms co-evolve within the context of their community as a composite of many species within the boundaries of ecological factors, which shape their microenvironment and control the direction of their evolution [4]. This concept was established early in microbial ecology, as the famous and frequently cited quote of Baas Becking L., stating that ‘Everything is everywhere, but, the environment selects’, which summarises the environment-dependent mechanisms of community assembly [67]. This is also true for pathogenic microbes, which are affected by the biotic and abiotic factors of their host plants, vectors, and surrounding microbes, all of which exert selective pressures. Such selective pressures drive the development of the pathobiome and evolution of adaptive mechanisms of pathogens to overcome the host defence; to enable dispersal inside and outside the host; and to facilitate vector adaptability, antagonism, and mutualism with the surrounding microbes [2].

An example of the co-evolution of mutualistic adaptative mechanisms is the bacterial endosymbionts of plant pathogenic fungi. The endosymbiont *Mycetohabitans rhizoxinica* undergoes genomic alteration by rearrangement and deletion of genomic information for adaptation to diverse habitats, and a large part of its horizontally acquired coding region is related to the biosynthesis of the virulence factor rhizoxin and harbours other genes involved in metabolic adaptation to intercellular life [60,68]. This interaction shifts from initial parasitism, where the bacterium is infectious to the fungus, to mutualism, where both benefit and successfully cause the disease [69]. The strong mutualism is maintained by endosymbiont-dependent host reproduction as a kind of treaty between the two partners, in which the bacterium provides a chemical weapon, rhizotoxin, to the fungus and the fungus, in turn, provides a powerful dispersal tool for the bacterium via its spores [60,69].

Evolutionary processes, such as gene flow and mutations are responsible for the emergence of novel genetic variants, representing the pathogenic agents equipped with weapons for survival and virulence to facilitate host colonisation and pathogenicity. These virulence-related weapons, such as different types of secretion systems and the ability to produce phytotoxins, virulence-related enzymes, and exopolysaccharides, differentiate the pathobiome from other commensal or symbiotic microbes [70]. Even within the same group of bacteria, the presence or absence of genetic material responsible for pathogenicity can differentiate pathogenic from beneficial plant-associated members. The bacterial group *Burkholderia sensu lato* is a powerful example in which genetically close bacterial species could be pathogenic or beneficial based on the presence of evolutionarily driven changes in their genetic material, such as the presence of pathogenicity-related genomic islands in the phytopathogenic members [68]. These genomic islands are foreign DNA regions that can be horizontally transferred—an evolutionary mechanism amongst bacteria—and integrated into pathogenic variants, endowing them with several specific accessory functions [71]. In addition, the diseased tissues with altered characteristics are ideal for colonisation by specific microbes associated with the disease. Although such microbes do not initiate pathogenesis, they can facilitate the development of symptoms of even a pre-existing condition [72]. These mechanisms shape the microbial and genetic composition along with the functional capacity of the microbial communities, including the pathobiome assemblage [2].

## 6. Case Studies of Model Diseases Studied from the Pathobiome Perspective

### 6.1. Acute Oak Decline from the Perspective of the Pathobiome

Acute oak decline is probably one of the few clear examples of a model disease studied from the perspective of the pathobiome paradigm. It is a complex decline threatening the native oak populations in the United Kingdom, and its incidence and distribution are increasing, with similar declines reported in other countries [73,74]. The disease is mediated by predisposing abiotic factors, such as temperature and rainfall, and contributing biotic factors, such as insects and bacteria, which together represent the disease complex [75]. The disease may be caused by the interaction amongst a polymicrobial complex of specific bacterial species, including *Brenneria goodwinii*, *Gibbsiella quercinecans*, and *Rahnella victoriana*, which are abundant in diseased tissues and produce virulence factors that cause typical disease symptoms [76].

The emergent properties of the acute oak decline pathobiome virulence have been speculated to be caused by a combination of the host–microbiota–insect interactions [75]. Inoculation with an isolate of a single bacterial species involved failed to produce disease lesions, whereas co-inoculation in the presence of the bark-boring beetle *Agrilus biguttatus* led to augmented expression of the bacterial virulence-related genes and development of typical symptoms. This result confirmed the hypothesis that complex host–pathobiome–insect interactions are essential for disease development [75]. With a contemporary approach using modified Koch’s postulates to investigate the causal complex pathobiome, *Brenneria goodwinii* and *Gibbsiella quercinecans* were confirmed to be necrotic to oak tissues, and together with the beetle larvae, they caused typical disease symptoms [73]. Meanwhile, the rhizosphere microbiome and physicochemical properties were associated with tree health and could protect the trees from the decline [77]. This integrated knowledge regarding the acute oak decline pathobiome has enabled remarkable advances in disease management in the United Kingdom.

### 6.2. Pine Wilt Disease from the Perspective of the Pathobiome

The continuously interacting pathogen–host complexes may sometimes make it difficult to determine the root cause of pathogenesis, which further supports the limitations and insufficiency of the traditional model based on the one pathogen–one disease dogma [72]. In pine trees, for instance, a serious disease causing devastating economic and environmental losses—pine wilt disease (PWD)—was originally thought to be caused by a single pathogen—the pinewood nematode (PWN) *Bursaphelenchus xylophilus* [78]. However, with accumulation of knowledge on the biotic factors and pathophysiology of PWD, fundamental pathogenicity-related roles of other key players, such as *Monochamus* beetles as the insect vector of the nematode, nematode-inhabiting microbes, and the ophiostomatoid blue stain fungus, in disease development and symptom exacerbation were revealed [79].

PWN is specifically associated with the vector pine sawyer longhorn beetle *Monochamus* sp., which transmits PWN from dead pine trees to the susceptible ones [80]. Growing evidence has indicated the occurrence of horizontal gene transfer in PWN from the associated bacteria and fungi, through which PWN acquires extended survival ability and pathogenicity [81,82]. A gene cluster encoding cellulose-degrading and other catabolic enzymes are phenotypically expressed by the distinct mode of parasitism of PWN on pine trees from that of other plant parasitic nematodes in terms of the diversity of food sources, such as plant tissues and *Ophiostoma* fungal species, and adaptability to various habitats, such as the insect vector tracheae or the resin canals of pine trees [82,83]. The PWN–fungus interaction is another intriguing biotic factor that positively affects PWN population, promoting PWD progression, and could therefore be considered another key player in the PWD pathobiome [80]. The presence of specific fungal species, such as *Ophiostoma minus* and *Sporothrix* sp. 1 promotes PWN proliferation, leading to further dispersal of large populations by the insect vector [84,85]. The insect vectors inoculated with *Ophiostoma minus* carried significantly more nematodes than the uninoculated ones. The diacetone alcohol fragrance emitted from pinewood infected by *Sporothrix* sp. 1 promoted insect vector growth and enhanced PWN fecundity [85].

Studies have also demonstrated that numerous bacterial species are associated with the affected pine tissues and the cuticular surface of PWN, based on scanning electron microscopic observation of widely distributed bacterial masses in cavities made by nematodes in the affected tissues for movement in the wood by the destruction of the wood tissues [86]. The PWN microbiome has been reported to assist the nematode in surviving and adapting to the toxic environment inside the tree by the detoxification of xenobiotics, such as α-pinene, which is a major compound in pine resin [87]. Moreover, the bacteria associated with the PWN cuticular surface, specifically the extreme oxidative stress-tolerant *Serratia* spp., have been reported to promote nematode survival under oxidative stress induced by pine as a defence mechanism [88].

Along with the ecological functions of the PWN microbiome that promote PWN growth and survival, bacteria associated with the nematode have also been suggested to play key roles in its pathogenicity [80]. This speculation was originally based on the observation of histological and physiological changes in the affected pine trees before the rapid increase in PWN numbers [89]. Nematode-carried bacteria have been reported to directly contribute to the development of disease symptoms by producing a major virulence factor. Inoculation of surface-sterilised axenic (microbe-free) nematodes did not induce wilting and browning symptoms in pine seedlings, but co-inoculation with certain bacterial species did so [90]. Previous studies have reported that bacteria carried by the nematode produce phytotoxins, which play important roles in PWD development. Pyochelin produced by *Burkholderia arboris*, cyclic dipeptides produced by *Pseudomonas fluorescens*, and phenylacetic acid produced by *Bacillus* spp., all of which are associated with PWN, are phytotoxic to pine seedlings and are the major virulence factors in disease development [91,92,93].

In addition to the roles of microbes in PWD pathogenesis described in this model, the endophytic, root, and rhizophere microbes associated with pine trees play important roles in supporting plant growth and inducing plant defence [94,95,96]. Characterisation of the pine tree microbiome predicted the presence of several functional orthologs related to the promotion of plant growth- and defence-related traits, such as expression of chitinases [95]. Hence, the rich community of pine endophytic microbes represents a source of functional traits that could be utilised for PWD management. Kim et al. [97] isolated three endophytic bacterial species that could induce systemic resistance against PWD and suggested these as potential biocontrol alternatives for disease management. In the light of these findings, studying PWD from the perspective of the pathobiome paradigm would allow for designing treatments involving the manipulation of the niche, which supports the development of the primary and secondary etiological agents of the disease. Studies on the roles of individual species might be limited in explaining the complexity of the disease mechanism and should therefore extend beyond the direct host–pathogen interaction to the host–niche–pathobiome interaction, and selective forces guiding the assembly of the associated microbes should be considered [2].

## 7. Conclusions

Advanced analytical tools in metagenomics, meta-transcriptomics, and meta-proteomics, combined with metabolomic analyses, to investigate the composition and functions of the plant microbiome have improved our understanding of the assemblage of and interactions amongst plant-associated microbes. Recent studies have indicated that the classic one pathogen–one disease hypothesis is insufficient to explain the disease process, and the field of plant pathology is currently witnessing a steady transition from this classic concept to the pathobiome paradigm. The transition into systems biology, particularly pathobiome research, is still in an early stage, and such studies require highly advanced facilities and tools, as well as a close collaboration between plant pathologists and ecologists. However, the presence of several diseases and the emergence of new ones, which cannot be effectively managed using classic plant pathological approaches, such as woody tree decline or wilt, warrant rapid advances in pathobiome research to find timely solutions for such threats. It is also worth mentioning that the one pathogen–one disease dogma remains a basic approach for investigation of most diseases, and will benefit from the recent advancements offered by the multi-omics approaches in the understanding of the plant pathogens within their biotic and abiotic environment. The knowledge of plant pathogens within their biotic and abiotic environments is anticipated to shape the future of plant disease management by optimising the conducive conditions against the formation of the pathobiome and offering a holistic approach to control plant diseases.

## Figures and Tables

**Table 1:** Examples of the different types of interactions among plant pathogenic species.

Interacting Species	Host Plant and Disease	Type of Interaction
*Burkholderia glumae* ↔ *Fusarium graminearum*	Rice panicle and seedling blight—*Fusarium* head blight	Bacteria-fungi co-operative interaction
*Mycetohabitans rhizoxinica* ↔ *Rhizopus microsporus*	Rice seedling blight	Bacteria-fungi endosymbiotic mutualism
*Albugo candida* ↔ *Phytophthora infestans*	Crucifers, downy or powdery mildew and *Phytophthora* blight	Fungi-fungi co-operative interaction
*Burkholderia gladioli* ↔ *Burkholderia glumae* and *Burkholderia plantarii*	Rice panicle and seedling blight	Bacteria-bacteria antagonism

## Data Availability

Not applicable as there are no additional data for this review.

## References

[1] Bettenfeld P., Fontaine F., Trouvelot S., Fernandez O., Courty P.E. (2020). Woody Plant Declines. What’s wrong with the Microbiome?. Trends Plant Sci..

[2] Vayssier-Taussat M., Albina E., Citti C., Cosson J., Jacques M.-A., Lebrun M.-H., Le Loir Y., Ogliastro M., Petit M.-A., Roumagnac P. (2014). Shifting the paradigm from pathogens to pathobiome: New concepts in the light of meta-omics. Front. Cell Infect. Microbiol..

[3] Baltrus D.A. (2017). Adaptation, specialization, and coevolution within phytobiomes. Curr. Opin. Plant Biol..

[4] Little A.E., Robinson C.J., Peterson S.B., Raffa K.F., Handelsman J. (2008). Rules of engagement: Interspecies interactions that regulate microbial communities. Annu. Rev. Microbiol..

[5] Gordon J., Youle M., Knowlton N., Rohwer F., Relman D.A. (2013). Superorganisms and Holobionts. Microbe.

[6] Bai Y., Müller D.B., Srinivas G., Garrido-Oter R., Potthoff E., Rott M., Dombrowski N., Münch P.C., Spaepen S., Remusemsermann M.N.P. (2015). Functional overlap of the Arabidopsis leaf and root microbiota. Nat. Cell Biol..

[7] Berg G., Rybakova D., Grube M., Köberl M. (2016). The plant microbiome explored: Implications for experimental botany. J. Exp. Bot..

[8] Rolli E., Marasco R., Vigani G., Ettoumi B., Mapelli F., DeAngelis M.L., Gandolfi C., Casati E., Previtali F., Gerbino R. (2015). Improved plant resistance to drought is promoted by the root-associated microbiome as a water stress-dependent trait. Environ. Microbiol..

[9] Vandenkoornhuyse P., Quaiser A., Duhamel M., Le Van A., Dufresne A. (2015). The importance of the microbiome of the plant holobiont. New Phytol..

[10] Scholthof K.-B.G. (2006). The disease triangle: Pathogens, the environment and society. Nat. Rev. Genet..

[11] Ogoshi A., Sneh B., Jabaji-hare S., Neate S., Dijst G. (1996). Introduction—The genus Rhizoctonia. Rhizoctonia Species: Taxonomy, Molecular Biology, Ecology, Pathology and Disease Control.

[12] Wu J., Ma H., Lü M., Han S., Zhu Y., Jin H., Liang J., Liu L., Xu J. (2010). Rhizoctonia fungi enhance the growth of the endangered orchid Cymbidium goeringii. Botany.

[13] Buonaurio R., Moretti C., Da Silva D.P., Cortese C., Ramos C., Venturi V. (2015). The olive knot disease as a model to study the role of interspecies bacterial communities in plant disease. Front. Plant Sci..

[14] Hosni T., Moretti C., Devescovi G., Suarez-Moreno Z.R., Fatmi M.B., Guarnaccia C., Pongor S., Onofri A., Buonaurio R., Venturi V. (2011). Sharing of quorum-sensing signals and role of interspecies communities in a bacterial plant disease. ISME J..

[15] Da Silva D.P., Castañeda-Ojeda M.P., Moretti C., Buonaurio R., Ramos C., Venturi V. (2014). Bacterial multispecies studies and microbiome analysis of a plant disease. Microbiology.

[16] Marchi G., Sisto A., Cimmino A., Andolfi A., Cipriani M.G., Evidente A., Surico G. (2006). Interaction between Pseudomonas savastanoi pv. savastanoi and Pantoea agglomerans in olive knots. Plant Pathol..

[17] Kim N., Kim J.J., Kim I., Mannaa M., Park J., Kim J., Lee H.H., Lee S.B., Park D.S., Sul W.J. (2020). Type VI secretion systems of plantpathogenic *Burkholderia* glumae BGR1 play a functionally distinct role in interspecies interactions and virulence. Mol. Plant Pathol..

[18] Tian B.-Y., Cao Y., Zhang K.-Q. (2015). Metagenomic insights into communities, functions of endophytes and their associates with infection by root-knot nematode, Meloidogyne incognita, in tomato roots. Sci. Rep..

[19] Cocolin L., Mataragas M., Bourdichon F., Doulgeraki A., Pilet M.F., Jagadeesan B., Rantsiou K., Phister T. (2018). Next generation microbiological risk assessment metaomics: The next need for integration. Int. J. Food Microbiol..

[20] Pervaiz T., Lotfi A., Haider M.S., Haifang J., Fang J. (2017). High Throughput Sequencing Advances and Future Challenges. J. Plant Biochem. Physiol..

[21] Mercado-Blanco J., Abrantes I., Caracciolo A.B., Bevivino A., Ciancio A., Grenni P., Hrynkiewicz K., Kredics L., Proença D.N. (2018). Belowground Microbiota and the Health of Tree Crops. Front. Microbiol..

[22] Langille M.G.I., Zaneveld J., Caporaso J.G., McDonald D., Knights D., Reyes J.A., Clemente J.C., Burkepile D.E., Thurber R.L.V., Knight R. (2013). Predictive functional profiling of microbial communities using 16S rRNA marker gene sequences. Nat. Biotechnol..

[23] Sharpton T.J. (2014). An introduction to the analysis of shotgun metagenomic data. Front. Plant Sci..

[24] Broberg M., Doonan J., Mundt F., Denman S., McDonald J.E. (2018). Integrated multi-omic analysis of host-microbiota interactions in acute oak decline. Microbiome.

[25] Zheng T., Zhang K., Zhu X., Guan L., Jiu S., Li X., Nasim M., Jia H., Fang J. (2019). Integrated metatranscriptome and transcriptome reveals the microbial community composition and physiological function of xylem sap on grapevine during bleeding period. Genes Genom..

[26] Singh D.P., Prabha R., Gupta V.K., Verma M.K. (2018). Metatranscriptome Analysis Deciphers Multifunctional Genes and Enzymes Linked With the Degradation of Aromatic Compounds and Pesticides in the Wheat Rhizosphere. Front. Microbiol..

[27] Xu T., Lei L., Shi J., Wang X., Chen J., Xue M., Sun S., Zhan B., Xia Z., Jiang N. (2019). Characterization of maize translational responses to sugarcane mosaic virus infection. Virus Res..

[28] Asselin J.E., Lin J., Perez-Quintero A.L., Gentzel I., Majerczak D., Opiyo S.O., Zhao W., Paek S.M., Kim M.G., Coplin D.L. (2015). Perturbation of maize phenylpropanoid metabolism by an AvrE family type III effector from *Pantoea stewartii*. Plant Physiol..

[29] Castro-Moretti F.R., Gentzel I., Mackey D., Alonso A.P. (2020). Metabolomics as an Emerging Tool for the Study of Plant-Pathogen Interactions. Metabolites.

[30] Luna E., Pastor V., Robert J., Flors V., Mauch-Mani B., Ton J. (2011). Callose Deposition: A Multifaceted Plant Defense Response. Mol. Plant Microbe Interact..

[31] Zhang H., Sonnewald U. (2017). Differences and commonalities of plant responses to single and combined stresses. Plant J..

[32] Denancé N., Sánchez-Vallet A., Goffner D., Molina A. (2013). Disease resistance or growth: The role of plant hormones in balancing immune responses and fitness costs. Front. Plant Sci..

[33] Zhu L., Zhou Y., Li X., Zhao J., Guo N., Xing H. (2018). Metabolomics Analysis of Soybean Hypocotyls in Response to Phytophthora sojae Infection. Front. Plant Sci..

[34] Apparecido R.D.P., Carlos E.F., Lião L., Vieira L.G.E., Alcantara G.B. (2017). NMR-based metabolomics of transgenic and non-transgenic sweet orange reveals different responses in primary metabolism during citrus canker development. Metabolomics.

[35] Tsuge T., Harimoto Y., Akimitsu K., Ohtani K., Kodama M., Akagi Y., Egusa M., Yamamoto M., Otani H. (2013). Host-selective toxins produced by the plant pathogenic fungus Alternaria alternata. FEMS Microbiol. Rev..

[36] Balendres M.A., Nichols D.S., Tegg R.S., Wilson C.R. (2016). Metabolomes of Potato Root Exudates: Compounds That Stimulate Resting Spore Germination of the Soil-Borne Pathogen Spongospora subterranea. J. Agric. Food Chem..

[37] Malinowski R., Nisler J., Borhan M.H., Spíchal L., Strnad M., Rolfe S.A. (2016). The role of cytokinins in clubroot disease. Eur. J. Plant Pathol..

[38] Lee D.-K., Ahn S., Cho H.Y., Yun H.Y., Park J.H., Lim J., Lee J., Kwon S.W. (2016). Metabolic response induced by parasitic plant-fungus interactions hinder amino sugar and nucleotide sugar metabolism in the host. Sci. Rep..

[39] Collemare J., O’Connell R.J., Lebrun M.-H. (2019). Nonproteinaceous effectors: The terra incognita of plant–fungal interactions. New Phytol..

[40] Keller N.P., Turner G., Bennett J.W. (2005). Fungal secondary metabolism—From biochemistry to genomics. Nat. Rev. Genet..

[41] Dallery J.F., Lapalu N., Zampounis A., Pigné S., Luyten I., Amselem J., Wittenberg A.H.J., Zhou S., de Queiroz M.V., Robin G.P. (2017). Gapless genome assembly of Colletotrichum higginsianum reveals chromosome structure and association of tranposable elements with secondary metabolite gene clusters. BMC Genom..

[42] Harris L.J., Balcerzak M., Johnston A., Schneiderman D., Ouellet T. (2016). Host preferential Fusarium graminearum gene expression during infection of wheat, barley, and maize. Fungal Biol..

[43] Weiberg A., Wang M., Lin F.-M., Zhao H., Zhang Z., Kaloshian I., Huang H.-D., Jin H. (2013). Fungal Small RNAs Suppress Plant Immunity by Hijacking Host RNA Interference Pathways. Science.

[44] Wang M., Weiberg A., Lin F.-M., Thomma B.P.H.J., Huang H.-D., Jin H. (2016). Bidirectional cross-kingdom RNAi and fungal uptake of external RNAs confer plant protection. Nat. Plants.

[45] Wang B., Sun Y., Song N., Zhao M., Liu R., Feng H., Wang X., Kang Z. (2017). *Puccinia striiformis f.* sp. *tritici* microRNA-like RNA 1 (*Pst*-milR1), an important pathogenicity factor of *Pst*, impairs wheat resistance to *Pst* by suppressing the wheat pathogenesisrelated 2 gene. New Phytol..

[46] De Fazio J., Fleming I.D., Shakhsheer B., Zaborina O., Alverdy J.C. (2014). The opposing forces of the intestinal Microbiome and the emerging pathobiome. Surg. Clin. N. Am..

[47] Bass D., Stentiford G.D., Wang H.-C., Koskella B., Tyler C.R. (2019). The Pathobiome in Animal and Plant Diseases. Trends Ecol. Evol..

[48] Pitlik S.D., Koren O. (2017). How holobionts get sick—Toward a unifying scheme of disease. Microbiome.

[49] Whitelaw-Weckert M.A., Rahman L., Appleby L.M., Hall A., Clark A.C., Waite H., Hardie W.J. (2013). Coinfection by Botryosphaeriaceae and *Ilyonectria* spp. fungi during propagation causes decline of young grafted grapevines. Plant Pathol..

[50] Moura M.L., Jacques L.A., Brito L.M., Mourao I.M., Duclos J. (2005). Tomato pith necrosis caused by *P. corrugata* and *P. mediterranea*: Severity of damages and crop loss assessment. Acta Hort..

[51] Canaday C.H., Wyatt J.E., Mullins J.A. (1991). Resistance to broccoli to bacterial soft rot caused by *Pseudomonas marginalis* and fluorescent Pseudomonas species. Plant Dis..

[52] Lamichhane J.R., Venturi V. (2015). Synergisms between microbial pathogens in plant disease complexes: A growing trend. Front. Plant Sci..

[53] Jung B., Park J., Kim N., Li T., Kim S., Bartley L.E., Kim J., Kim I., Kang Y., Yun K. (2018). Cooperative interactions between seed-borne bacterial and air-borne fungal pathogens on rice. Nat. Commun..

[54] Stopnisek N., Zühlke D., Carlier A., Barberán A., Fierer N., Becher D., Riedel K., Eberl L., Weisskopf L. (2015). Molecular mechanisms underlying the close association between soil *Burkholderia* and fungi. ISME J..

[55] Partida-Martinez L.P., Hertweck C. (2006). A Gene Cluster Encoding Rhizoxin Biosynthesis in “*Burkholderia* rhizoxina”, the Bacterial Endosymbiont of the Fungus Rhizopus microsporus. ChemBioChem.

[56] Takahashi M., Iwasaki S., Kobayashi H., Okuda S., Murai T., Sato Y. (1987). Rhizoxin binding to tubulin at the maytansine-binding site. Biochim. Biophys. Acta Gen. Subj..

[57] Scherlach K., Busch B., Lackner G., Paszkowski U., Hertweck C. (2012). Symbiotic cooperation in the biosynthesis of a phytotoxin. Angew. Chem..

[58] Partida-Martinez L.P., Hertweck C. (2005). Pathogenic fungus harbours endosymbiotic bacteria for toxin production. Nat. Cell Biol..

[59] Lackner G., Moebius N., Hertweck C. (2010). Endofungal bacterium controls its host by an hrp type III secretion system. ISME J..

[60] Lackner G., Moebius N., Partida-Martínez L.P., Boland S., Hertweck C. (2011). Evolution of an endofungal Lifestyle: Deductions from the *Burkholderia* rhizoxinica Genome. BMC Genom..

[61] Cooper A.J., Latunde-Dada A.O., Woods-Tör A., Lynn J., Lucas J.A., Crute I.R., Holub E.B. (2008). Basic compatibility of Albugo candida in Arabidopsis thaliana and Brassica juncea causes broad spectrum suppression of innate immunity. Mol. Plant Microbe Interact..

[62] Prince D.C., Rallapalli G., Xu D., Schoonbeek H.J., Çevik V., Asai S., Kemen E., Cruz-Mireles N., Kemen A., Belhaj K. (2017). Albugo-imposed changes to tryptophanderived antimicrobial metabolite biosynthesis may contribute to suppression of non-host resistance to Phytophthora infestans in Arabidopsis thaliana. BMC Biol..

[63] Kim N., Mannaa M., Kim J., Lee C., Kim S.M., Ra J.E., Lee H.H., Seo Y.S. (2020). The in vitro and in planta interspecies interactions among rice-pathogenic *Burkholderia* species. Plant Dis..

[64] Miyagawa H. (2000). Biocontrol of bacterial seedling blight of rice caused by *Burkholderia* gladioli using with its avirulent isolate. Jpn. J. Phytopathol..

[65] Riera-Ruiz C., Castro-Lara J., Jimenez-Feijoó M.I., Cevallos-Cevallos J.M. (2018). Interactions of *Burkholderia glumae* and *B. gladioli* in symptom development in rice seeds and seedlings. Can. J. Plant Pathol..

[66] Elshafie H.S., Racioppi R., Bufo S.A., Camele I. (2016). In vitro study of biological activity of four strains of *Burkholderia gladioli* pv. agaricicola and identification of their bioactive metabolites using GC-MS. Saudi J. Biol. Sci..

[67] De Wit R., Bouvier T. (2006). Everything is everywhere, but, the environment selects, what did Baas Becking and Beijerinck really say?. Environ. Microbiol..

[68] Mannaa M., Park I., Seo Y.-S. (2018). Genomic Features and Insights into the Taxonomy, Virulence, and Benevolence of Plant-Associated *Burkholderia* Species. Int. J. Mol. Sci..

[69] Partida-Martinez L.P., Monajembashi S., Greulich K.O., Hertweck C. (2007). Endosymbiont-dependent host reproduction maintains bacterial fungal mutualism. Curr. Biol..

[70] Hacker J., Kaper J.B. (2000). Pathogenicity Islands and the Evolution of Microbes. Annu. Rev. Microbiol..

[71] Juhas M., Van Der Meer J.R., Gaillard M., Harding R.M., Hood D.W., Crook D.W. (2009). Genomic islands: Tools of bacterial horizontal gene transfer and evolution. FEMS Microbiol. Rev..

[72] Rogers G., Hoffman L.R., Carroll M.P., Bruce K.D. (2013). Interpreting infective microbiota: The importance of an ecological perspective. Trends Microbiol..

[73] Denman S., Brown N., Kirk S., Jeger M., Webber J. (2014). A description of the symptoms of Acute Oak Decline in Britain and a comparative review on causes of similar disorders on oak in Europe. For. Int. J. For. Res..

[74] Ruffner B., Schneider S., Meyer J., Queloz V., Rigling D. (2020). First report of acute oak decline disease of native and non-native oaks in Switzerland. New Dis. Rep..

[75] Doonan J.M., Broberg M., Denman S., McDonald J.E. (2020). Host microbiota insect interactions drive emergent virulence in a complex tree disease. Proc. R. Soc. B.

[76] Denman S., Doonan J., Ransom-Jones E., Broberg M., Plummer S., Kirk S., Scarlett K., Griffiths A.R., Kaczmarek M., Forster J. (2018). Microbiome and infectivity studies reveal complex polyspecies tree disease in Acute Oak Decline. ISME J..

[77] Pinho D., Barroso C., Froufe H., Brown N., Vanguelova E., Egas C., Denman S. (2020). Linking tree health, rhizosphere physi-cochemical properties, and microbiome in acute oak decline. Forests.

[78] Tóth Á. (2011). *Bursaphelenchus xylophilus*, the pinewood nematode: Its significance and a historical review. Acta Biol. Szeged..

[79] Zhao L., Mota M., Vieira P., Butcher R.A., Sun J. (2014). Interspecific communication between pinewood nematode, its insect vector, and associated microbes. Trends Parasitol..

[80] Futai K. (2013). Pine wood nematode, Bursaphelenchus xylophilus. Ann. Rev. Phytopathol..

[81] Kikuchi T., Cotton J.A., Dalzell J.J., Hasegawa K., Kanzaki N., McVeigh P., Takanashi T., Tsai I.J., Assefa S.A., Cock P.J.A. (2011). Genomic Insights into the Origin of Parasitism in the Emerging Plant Pathogen *Bursaphelenchus xylophilus*. PLOS Pathog..

[82] Shinya R., Morisaka H., Takeuchi Y., Futai K., Ueda M. (2013). Making headway in understanding pine wilt disease: What do we perceive in the postgenomic era?. J. Biosci. Bioeng..

[83] Kikuchi T., Shibuya H., Jones J.T. (2005). Molecular and biochemical characterization of an endo-β-1,3-glucanase from the pinewood nematode *Bursaphelenchus xylophilus* acquired by horizontal gene transfer from bacteria. Biochem. J..

[84] Maehara N., Futai K. (1997). Effect of fungal interactions on the numbers of the pinewood nematode, *Bursaphelenchus xylophilus* (Nematoda: Aphelenchoididae), carried by the Japanese pine sawyer, Monochamus alternatus (Coleoptera: Cerambycidae). Fundam. Appl. Nematol..

[85] Zhao L., Lu M., Niu H., Fang G., Zhang S., Sun J. (2013). A native fungal symbiont facilitates the prevalence and development of an invasive pathogen-native vector symbiosis. Ecology.

[86] Mamiya Y. (2012). Scanning Electron Microscopy of Pine Seedling Wood Tissue Sections Inoculated with the Pinewood Nematode *Bursaphelenchus xylophilus* Previously Prepared for Light Microscopy. J. Nematol..

[87] Cheng X.Y., Tian X.L., Wang Y.S., Lin R.M., Mao Z.C., Chen N., Xie B.Y. (2013). Metagenomic analysis of the pinewood nematode microbiome reveals a symbiotic relationship critical for xenobiotics degradation. Sci. Rep..

[88] Vicente C.S., Ikuyo Y., Mota M., Hasegawa K. (2013). Pinewood nematode-associated bacteria contribute to oxidative stress resistance of *Bursaphelenchus xylophilus*. BMC Microbiol..

[89] Oku H., Shiraishi T., Ouchi S., Kurozumi S., Ohta H. (1980). Pine wilt toxin, the metabolite of a bacterium associated with a nematode. Naturwissenschaften.

[90] Han Z., Hong Y.D., Zhao B.G. (2003). A Study on Pathogenicity of Bacteria Carried by Pine Wood Nematodes. J. Phytopathol..

[91] Le Dang Q., Son S.W., Cheon H.-M., Choi G.J., Choi Y.H., Jang K.S., Lim C.H., Kim J.-C. (2011). Pyochelin isolated from *Burkholderia* arboris KRICT1 carried by pine wood nematodes exhibits phytotoxicity in pine callus. Nematology.

[92] Guo Q., Guo D., Zhao B., Xu J., Li R. (2007). Two Cyclic Dipeptides from Pseudomonas fluorescens GcM5-1A Carried by the Pine Wood Nematode and Their Toxicities to Japanese Black Pine Suspension Cells and Seedlings in vitro. J. Nematol..

[93] Kawazu K., Zhang H., Yamashita H., Kanzaki H. (1996). Relationship between the Pathogenicity of the Pine Wood Nematode, *Bursaphelenchus xylophilus*, and Phenylacetic Acid Production. Biosci. Biotechnol. Biochem..

[94] Proença D.N., Grass G., Morais P.V. (2016). Understanding pine wilt disease: Roles of the pine endophytic bacteria and of the bacteria carried by the disease-causing pinewood nematode. Microbiology.

[95] Alves M.S., Pereira A., Vicente C.S.L., Matos P., Henriques J., Lopes H., Nascimento F.X., Mota M., Correia A., Henriques I. (2018). The role of bacteria in pine wilt disease: Insights from microbiome analysis. FEMS Microbiol. Ecol..

[96] Mannaa M., Han G., Jeon H.W., Kim J., Kim N., Park A.R., Kim J.-C., Seo Y.-S. (2020). Influence of Resistance-Inducing Chemical Elicitors against Pine Wilt Disease on the Rhizosphere Microbiome. Microorganisms.

[97] Kim N., Jeon H.W., Mannaa M., Jeong S.I., Kim J., Kim J., Lee C., Park A.R., Kim J.C., Seo Y.S. (2019). Induction of resistance against pine wilt disease caused by *Bursaphelenchus xylophilus* using selected pine endophytic bacteria. Plant Pathol..

